# Acute Metabolic Changes with Lower Leg-Positioned Wearable Resistances during Submaximal Running in Endurance-Trained Runners

**DOI:** 10.3390/sports7100220

**Published:** 2019-10-11

**Authors:** Allister P. Field, Nicholas Gill, Aaron M. Uthoff, Dan Plews

**Affiliations:** 1 Sports Performance Research Institute New Zealand (SPRINZ) at AUT Millennium, Auckland University of Technology, Auckland 0632, New Zealand; allister.field@hotmail.co.nz (A.P.F.); nicholas.gill@nzrugby.co.nz (N.G.); daniel.plews@aut.ac.nz (D.P.); 2 Adams Centre for High Performance, University of Waikato, Tauranga 3116, New Zealand

**Keywords:** limb loading, heart rate, oxygen consumption

## Abstract

The aim of this study was to determine the acute metabolic effects of different magnitudes of wearable resistance (WR) attached to the lower leg during submaximal running. Fifteen endurance-trained runners (37.8 ± 6.4 years; 1.77 ± 0.7 m; 72.5 ± 9.8 kg; 58.9 ± 7.4 L/min VO_2max_; 45.7 ± 5.8 min 10 K run time) completed seven submaximal running trials with WR loads of 0, 0.5, 1, 1.5, 2, 2.5 and 3% body mass (BM). Based on regression data, for every 1% BM increase of additional load, oxygen consumption (VO_2_) increased by 2.56% and heart rate increased by 1.16%. Inferential based analysis identified that ≤1% BM were enough to elicit responses in VO_2_, with a possible small increase (effect size (ES), 90% confidence interval (CI): 0.22, 0.17 to 0.39), while 3% BM loads produced a most likely very large increase (ES, 90% CI: 0.51, 0.42 to 0.60). A training load score was extrapolated using heart rate data to determine the amount of internal stress. An additional 1% BM resulted in an extra 0.39 (0.29 to 0.47) increase in internal stress over five minutes. Lower leg WR elicited substantial increases in lactate production from the lightest loading (0.5% BM), with a likely moderate increase (ES, 90% CI: 0.49, 0.30 to 0.95). Lower-leg positioned WR provides a running-specific overload with loads ≥ 1% BM resulting in substantial changes in metabolic responses.

## 1. Introduction

From early generations as hunter gatherers, humans have evolved to run for extended periods of time, commonly referred to as endurance running [1]. Today, endurance running attracts millions of participants globally for both recreational and competitive purposes. Endurance running performance is determined by physiological mechanisms, such as maximum oxygen uptake (VO_2max_) [2], blood lactate concentrations relative to the percentage of VO_2max_ that a runner can sustain (%VO_2max_ at second ventilatory threshold VT_2_) [3,4], and the metabolic cost of running at a given velocity, i.e., running economy (RE) [2]. These factors may be modified through varying strength training methods, leading to improved RE, muscular power production and running performance [5]. It is, therefore, important for practitioners to understand the acute effects of training methods on the physiological determinants of endurance running to maximize training transference and effectively progress overload to optimize performance and minimize the risk of injury.

One training method which enables specific overload to be applied during training is the use of wearable resistance (WR) [6]. With WR, external loads can be affixed to distal segments of the body while runners participate in their normal running training [6,7]. Adding WR to the limbs using loads ranging from 0.3–8.5% BM has shown a greater increase in metabolic demand compared to unloaded running, indicated by increases in oxygen consumption (V̇O_2_), heart rate (HR) and internal stress [6,8]. Additionally, metabolic demands increase when comparable loads are placed more distal [9,10,11]. For example, Martin [11] found that adding 0.50 (0.69% BM) and 1.0 kg (1.39% BM) to each ankle at a running velocity of 12 km·h^−1^ substantially increased V̇O_2_ by 3.3% (ES = 0.56) and 7.2% (ES = 1.20) respectively, yet Field et al. [8] concluded that loads ≥ 3% BM were required to elicit substantial responses in V̇O_2_ in endurance-trained runners when using more proximally located thigh-positioned WR at self-paced speeds on a 1% motorized treadmill (4.3–8.1%; ES = 0.24–0.43). Moreover, HR increases have been found to be consistent with increases in V̇O_2_ [8,10,11].

While WR appears to affect the physical determinants of endurance running [11], it is unknown what the metabolic load–response relationship looks like with incremental WR lower-limb loading. Understanding the metabolic effects between loads may enable practitioners to more accurately prescribe loads to target different aspects of training. Therefore, the purpose of this research was to examine the acute metabolic effects of submaximal running with WR loads ranging from 0.5%–3% BM attached to the lower legs and establish a load–response relationship to estimate increases in metabolic cost with increasing WR in endurance-trained runners. It was hypothesized that the additional loading from the WR would increase our primary outcome measure, acute metabolic response, i.e., oxygen consumption (VO2) and HR, during submaximal running as a result of overloading the leg musculature. For our secondary outcome measures, we also hypothesized that there would be increased responses in lactate, internal stress and perceived exertion associated with lower leg loading.

## 2. Materials and Methods

### 2.1. Subjects

Fifteen endurance-trained runners with an average VO_2max_ of 58.9 ± 7.4 L/min and 10 K running time of 45.7 ± 5.8 min (four female and 11 male; 37.8 ± 6.4 years; 1.77 ± 0.7 m; 72.5 ± 9.8 kg) were recruited to participate in this study. All runners had no history of any major health issues 12 months prior to commencement of the study and had completed a minimum of one-half marathon distance in the last 12 months. In addition, they were required to be actively involved in endurance-run training at the commencement of the study and had a minimum V̇O_2max_ of 50 and 40 mL·kg^−1^·min^−1^ for males and females, respectively. Ethical approval (17/172) for this study was obtained from the Auckland University of Technology Ethics Committee. Before testing, all participants provided informed consent in writing and completed a pre-exercise health questionnaire (Par-Q). 

### 2.2. Procedure 

**Figure 1:**
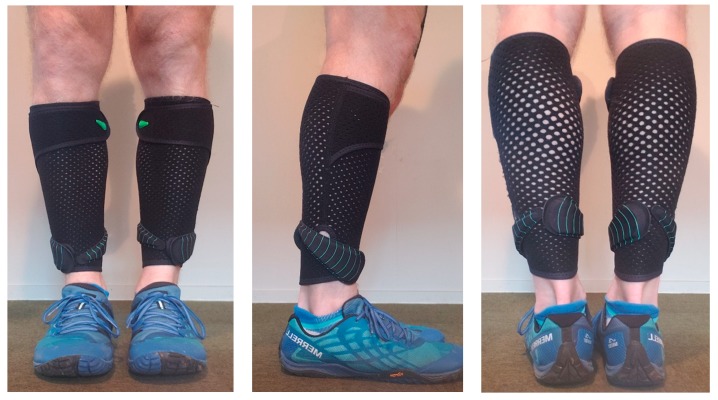
Example of lower-leg wearable resistance loading pattern (0.5% BM) for a 70 kg runner.

**Figure 2:**
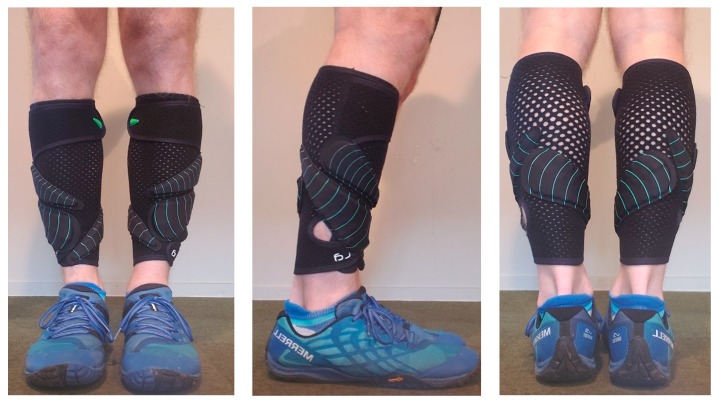
Example of lower-leg wearable resistance loading pattern (1.5% BM) for a 70 kg runner.

**Figure 3:**
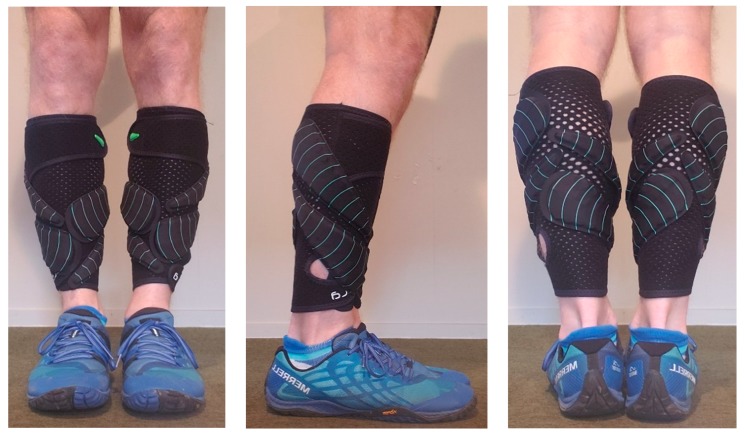
Example of lower-leg wearable resistance loading pattern (2.5% BM) for a 70 kg runner.

All running trials were conducted under stable laboratory conditions (ambient conditions: 21 ± 3 °C, <60% relative humidity) on a motorized treadmill (Woodway, Waukesha, WI, USA) with the gradient set a 1% [12]. A carbon dioxide and oxygen analyzer (Metalyzer Cortex, Biophysik GmbH, Leipzig, Germany) which was calibrated before each testing session according to the manufacturers specifications was used to quantify oxygen consumption, heart rate response data was collected using a heart rate (HR) monitor (Polar A300, Guangzhou, China). Lactate accumulation (La) was measured using a blood La analyzer (La Pro 2, Shiga, Japan) and all capillary samples were drawn from the preferred finger of the runner. Subjective data was measured through the rate of perceived exertion (RPE) using a modified BORG 10-point scale [13]. For the WR conditions, each loaded trial required participants to wear a pair of compression lower leg sleeves with associated loads (Lila^TM^, Exogen^TM^, Wilayah Persekutuan Kuala Lumpur, Malaysia). Weighted panels were in either 50, 100 or 200 g increments and total load for each trial was rounded to the nearest 50 g, as seen in Figure 1, Figure 2 and Figure 3. The loading strategy began with each WR load placed alternatively from the anterior to the posterior, in a stacked balance of distal to proximal. 

**Figure 4:**
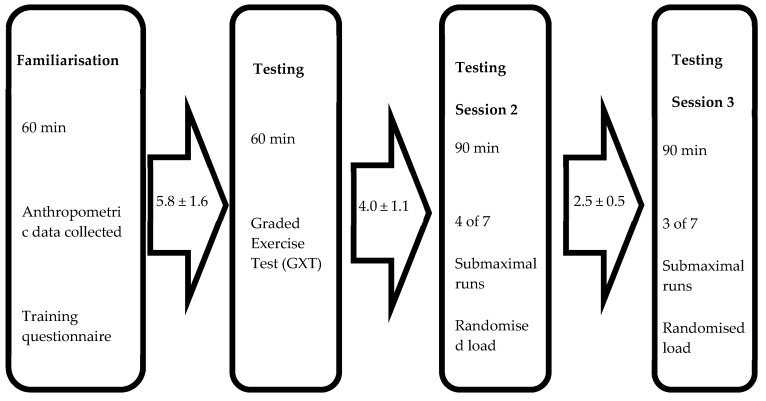
Structure of testing sessions.

Each participant was assessed over a maximum of a 15-day period. During this time, each participant was involved in one familiarization session and three testing sessions (see Figure 4). The purpose of the familiarization session was to acclimate the runner to the testing environment, i.e., treadmill running while wearing both the compressive lower-leg sleeves and all metabolic measuring equipment. Participants started by completing a self-paced run for 20-min followed by a 10-min recovery. During the recovery period, the graded exercise test (GXT) protocol was discussed and a HR monitor and gas mask were fitted. Participants then completed a further 10-min run following a GXT incremental protocol to become accustomed with the procedures, no data was collected. At the completion of the familiarization session, participants were instructed to refrain from training on the day of testing and any strenuous exercise or training 24 h prior to the testing sessions. Participants were asked to maintain their normal dietary intake and were given a 24-h training and food diary to keep themselves prior to completing the first round of loaded trials. They were asked to replicate this 24-h training and food diary 24 h before completing the second round of loaded trials. 

Testing session one occurred within 5.8 ± 1.6 days of completing the familiarization session. The purpose of session one was to establish VT_1_, VT_2_ and VO_2max_ and generate a V̇O_2_ response profile to graded exercise. Participants completed a self-paced 20-min warm-up on a treadmill and were given a recovery period of 10-min prior to the commencement of the GXT. Starting speed was maintained for 1-min followed by an increase of 0.5 km h^−1^ every 30 s until voluntary exhaustion [14]. Starting speed was adjusted on an individual basis to ensure volitional exhaustion at 8–12 min. Oxygen consumption was tracked continuously at a sampling rate of 0.1 Hz, HR and RPE recorded at each speed increment, with La being measured immediately following the test. V̇O_2max_ was an average over 30 s and was determined to be achieved if any one of the following criteria were met: a plateau in V̇O_2_ was reached despite an increase in workload, a respiratory exchange ratio (RER) > 1.15 was identified, a HR within five beats of age predicted maximum (220−Age) was reached or a peak exercise blood La concentration > 8 mmol/L was reached [15]. Testing sessions two and three served to measure metabolic and subjective responses during self-paced submaximal running trials, where velocity was matched and WR was the only variable altered. No order effect was identified for either testing session two or three (*p* = 0.82 and *p* = 0.83, respectively). Testing session two occurred within 4.0 ± 1.1 days of testing session one and testing session three occurred within 2.5 ± 0.5 days of testing session two to minimize fatigue between sessions. Testing session two included four wearable loads and testing session three included the final three wearable loads, load order was randomized and different for each runner. At the start of testing session two and three, an 8-min warm up set at a running speed equivalent to V̇T_1_ was completed, followed by a 10-min recovery. VT_1_ was chosen as this is close to the typical training intensity in endurance sports, in line with the polarized model of training. The polarized training model has been shown to be common practice among elite endurance runners, for whom slow long-distance training at lower intensities (<VT_2_) makes up 75% of an individual’s training volume, with shorter-, higher-intensity bouts of effort (>VT_2_) making up the remainder of the training program [13]. Each submaximal running trial lasted 5-min with 10-min of passive recovery between each subsequent trial. Oxygen consumption and HR were tracked for 2-min prior to each trial starting, for the 5-min of each trial (final 2-min used for analysis) and for 2-min post trial. Rate of perceived exertion and La was recorded immediately after completing each 5-min trial.

### 2.3. Statistical Analysis

The statistical aim of this study was to make an inference about the impact on metabolic stress of submaximal running with WR, which requires determining the magnitude of an outcome. Given the study design and practical importance, a magnitude-based inference approach was used for analysis to provide an indication of the amount and direction of change to the variables of interest [16]. Inferential statistics take into consideration the magnitude for the effect of interest and provide a practically meaningful interpretation of the data [17,18]. Accordingly, inferential statistics were used to examine the practical meaning of the observed changes in metabolic cost (V̇O_2_, HR, La) and perception (RPE) of submaximal running with load compared to unloaded similar to the approach used by Field et al. [8]. Confidence intervals (CI) were set at 90%. Data was presented as mean (SD) for each variable with corresponding effect size (ES) and mean/percent differences (90% CI) throughout. The smallest worthwhile change was used to determine if any observed changes were considered trivial, possible or likely, including the magnitude of each change, calculated as a change in score standardized to 0.2 of the between-subject SD from the unloaded condition [19]. The qualitative probabilities were defined by the scale <0.5% most likely trivial increase, <5% very likely trivial increase, <25% likely trivial increase, 25–75% possible small increase, >75% likely moderate increase, >95% very likely large increase, >99.5% most likely very large increase and the outcome was deemed unclear where the 5% and 90% CI of the mean change overlapped both the positive and negative outcomes [16]. To help quantify the internal load of WR based on relative exercise intensity and duration, a HR-based training impulse and session RPE were used to extrapolate a training stress score (TSS) for each load [20,21] for 10-min of running using Training Peaks^TM^ software (Training Peaks 3.0, Boulder, CO, USA). To understand the relationship between metabolic variables (V̇O_2_, HR and TSS) and load, a scatterplot was created in excel to establish a linear equation and R^2^ value for each variable. Formula used for calculating TSS [21]:

TSS = (sec × HR × IF)/(V̇T2 × 3600) × 100

IF (impact factor) = HR/V̇T2

Key: TSS: Training load score; HR: Heart rate (average heart rate during exercise); IF: Impact factor; V̇T2: Second ventilatory threshold (point at which lactate accumulation exceeds clearance).

## 3. Results

### Metabolic Responses

**Figure 5:**
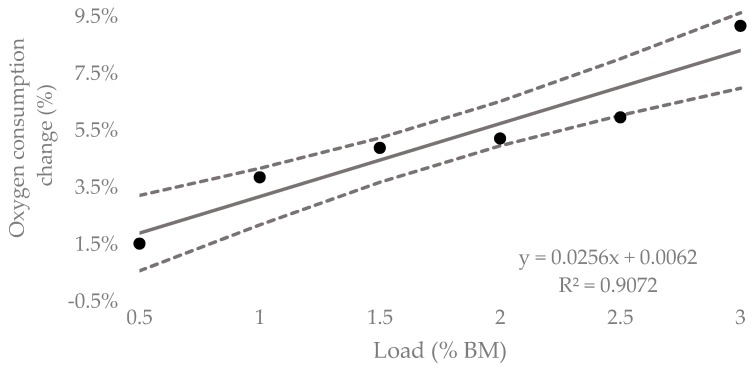
Mean percent increase in acute oxygen consumption to lower-leg WR for 5-min submaximal running trials compared to unloaded (90%CI).

Table 1 contains the means, standard deviations and custom effects as standardized units (ES ± 90% CI) for the acute oxygen responses for all loading conditions. The mean oxygen consumption of submaximal running at 0.5% BM resulted in a greater response, with a very likely trivial increase of 1.5% (0.16% to 3.22%). A possibly small increase in V̇O_2_ (3.9%, −1.87% to 5.86%), was seen at 1% BM. Both 1.5% and 2% BM resulted likely in moderate increases (4.9%, 3.1% to 6.73% and 5.2%, −3.53% to 6.92%, respectively) in mean V̇O_2_ response. The 2.5% BM generated a mean V̇O_2_ response of 6.0% (−3.98% to 7.96%) and resulted in a very likely large increase. The 3% BM generated a 9.2% (−7.62% to 10.7%) increase in mean V̇O_2_ response, which was reported to be most likely very large. Figure 5 contains the percentage change in oxygen response from unloaded to loaded (90% CI). Linear regression was carried out and showed a positive relationship (R^2^ = 0.91), representing an additional 2.56% (1.69 to 3.46%) increase in oxygen consumption for every 1% BM of additional load.

**Figure 6:**
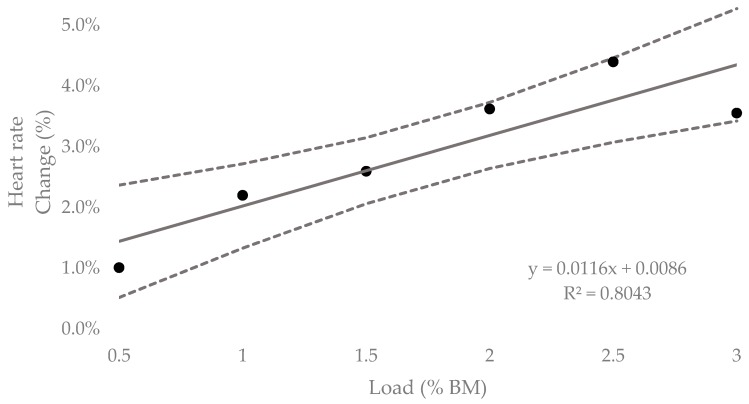
Percent increase in acute heart rate response to lower-leg loaded WR for 5-min submaximal running trials compared to unloaded (± 90% CI).

**Figure 7:**
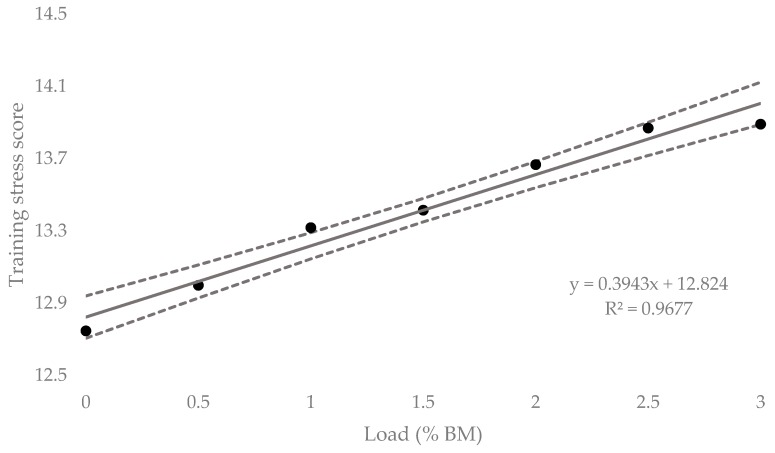
Extrapolated Training Load Score (TSS) for lower-leg loaded WR for the equivalent of 10-min of running (± 90% CI).

Table 2 contains the means, standard deviations and custom effects as standardized units (ES, 90% CI) for the acute HR responses for all loading conditions. The mean HR response at 0.5% BM resulted in a possible small increase of 1.0% (0.30 to 2.33%) from baseline. Both 1% and 1.5% BM resulted in likely moderate increases from 2.2% (−0.70% to 3.70%) and 2.6% (−1.43% to 3.77%), respectively. Most likely, large increases were found for 2%, 2.5% and 3% BM with mean HR responses equating to a 3.6% (−2.43% to 4.82%), 4.4% (−3.30% to 5.50%), and 3.6% (−2.09% to 5.02%) increase, respectively. Figure 6 contains the percentage change in HR response from unloaded to loaded (90% CI). Linear regression showed a positive relationship (R^2^ = 0.80), representing an additional 1.16% (0.58% to 1.77%) increase in HR response for every 1% BM of additional load. Figure 7 represents the relationship between the TSS extrapolated from collected HR data for the equivalent of 10-min of running at V̇T1 and load. The regression equation showed a positive linear relationship (R^2^ = 0.97), representing an additional 0.39 (0.29 to 0.47) of internal training stress for every 1% BM of additional load. 

Table 3 contains the means, standard deviations and custom effects as standardised units (ES, 90% CI) for the acute La responses for all loading conditions. Post-submaximal running with a load of 0.5% BM produced a mean La response 25.9% (−8.73% to 43.1%) greater than unloaded running, which was deemed a likely moderate increase. A very likely large increase in mean La of 30.5% (−14.25% to 46.8%) was associated with 1% BM. Both 1.5% and 2% BM resulted in likely moderate increases of 24.9% (−6.46% to 43.3%) and 29.0% (0.29% to 58.4%), respectively, from baseline. At 2.5% and 3% BM, mean La increases of 49.0% (−24.4% to 73.6%) and 54.6% (−29.5% to 79.6%) were, respectively, reported to be most likely very large.

Table 4 contains the means, standard deviations and custom effects as standardized units (ES, 90% CI) for the acute RPE responses for all loading conditions. With a load of 0.5% BM, a mean RPE increase from baseline resulted in a likely moderate increase. Both 1% and 1.5% BM resulted in very likely large increases from unloaded running. At 2%, 2.5% and 3% BM, most likely very large increases in mean RPE were generated compared to baseline.

## 4. Discussion

The aim of this study was to understand the acute metabolic effects of lower-leg WR during submaximal running in endurance-trained runners. Based on the regression data, it was determined that for every 1% BM of additional load, there is an expected 2.56% and 1.16% increase in V̇O_2_ and HR response, respectively. Inferential-based analysis demonstrated that loading of at least 1% BM was needed to have a possible small increase (3.86%) in V̇O_2_ response, with a most likely very large increase (9.18%) at 3% BM. The smallest loaded trial (0.5% BM) was enough to have a possible small increase (1.01%) in HR response. A TSS from the collected HR data was able to be extrapolated to establish the impact that additional load would have on a training session. This resulted in a predicted 0.39 increase in internal stress for every 1% BM of additional load for 10-min of loaded running at a speed equivalent to V̇T1. 

The V̇O_2_ and HR data collected in this study agrees with data previously reported, indicating that limb loading during submaximal running can increase metabolic cost compared to unloaded running [9,10]. This may be expected as limb loading has been shown to significantly (*p* < 0.05) increase both relative V̇O_2_ and HR responses in runners [10]. At running speeds ranging from ~12–14 km·h^−1^, oxygen consumption has been seen to increase by between 3.5% and 4.5% with each additional kg of load in untrained and endurance-trained runners [10,11,22]. The present study reported that for an additional load of 0.5%, 1%, 1.5%, 2%, 2.5% and 3% BM, a mean increase in V̇O_2_ of 1.5%, 3.9%, 4.9%, 5.2%, 6.0% and 9.2% was found, respectively. Comparatively, an increase in V̇O_2_ of 2.56% for every 1% BM (equivalent to 0.73 kg when extrapolated from the mean weight of participants) of additional load was also noted based on the linear regression equation. Accordingly, the increase in metabolic cost is less than previously reported; however, differences may be expected as the previous studies used loads placed more distally than those in the present study and it has been determined that comparative load moved more distal on the lower limb has a significantly (*p* < 0.05) greater impact on the metabolic cost of running [11]. However, when compared to the previous research using similar methods and population, the results of this study suggest that 1% BM increases in lower-leg-positioned WR has a 62% greater effect on VO_2_ compared to 1% BM increases in thigh-positioned WR [8]. 

In terms of HR responses, a similar trend to that of V̇O_2_ has previously been reported with slight increases due to additional load placed on the feet [10,11]; although, these researchers suggested that HR is a less sensitive measure of lower-limb loading. Recently, a study investigating the effects of thigh-positioned WR concluded that for every 1% increase in WR attached to the thighs, there was a 0.63% increase in HR, and that at least 2% BM loads were required to produce a possible small response [8]. Comparatively, the current study reported an increase in HR of 1.16% for every 1% BM (equivalent to 0.73 kg when extrapolated from the mean weight of participants) of additional load, which is less than half that of V̇O_2_ (2.56%) for the same load. Inferential-based analysis of the present study demonstrated that the smallest lower-leg WR load of 0.5% BM could produce a possible small increase (ES = 0.13; 1.01%) in HR response. 

Using the HR data collected, a TSS was extrapolated to help quantify the amount of internal stress each loaded trial would have over a 10-min running period, as it is a commonly used method for calculating training stress for endurance athletes. Based on the linear regression equation produced for TSS plotted against load, for every 1% BM of additional load, there is an extra 0.39 (± 0.06) increase in internal stress. That is over twice as much as has previously been reported for the same relative increase in load with thigh-positioned WR [8]. Practically, this means that by adding 1% BM to the lower legs, the running load increases by 3.86% over 5-min. As the interplay between exercise intensity and duration is an important factor in determining physiological adaptations in response to a training stimulus [23], loading the lower legs may provide a specific training method to either increase training load by adding weight and maintaining exercise duration, or by maintaining training load by using lower-leg WR with reduced training durations. In essence, lower-leg WR may provide a time-saving method to induce similar changes associated with long-duration training. However, the training effects of lower-leg WR are not currently scientifically established. Therefore, practitioners should be cognizant to adjust intensity or duration to minimize any adverse effects which occur as a result of increased acute training load. 

When using WR for endurance-trained athletes, it is important to understand how placement may influence performance, both positively, and potentially, negatively. This study showed that loading the lower leg can lead to acute metabolic changes which may have long-term training implications for endurance-trained athletes. Furthermore, compared to previous studies, it appears that lighter loads can be used on the lower legs to induce metabolic responses and effectively overload the muscles across the knee and the hip compared to loading the thighs, which would primarily overload the muscles of the hip. Readers should be cognizant that the use of magnitude-based inferences has been determined to inflate the potential for type I error [24]. Although, it should be recognized that confidence intervals alone or in conjunction with a *p*-value does not overtly address the question of clinical, practical, or mechanistic importance of an outcome [18]. Though it is unknown whether lower-leg-positioned WR has adverse effects on running mechanics, it is critical to consider that more distally placed WR may accentuate poor running mechanics than proximally located WR. Therefore, the authors recommend that particular attention be given to running technique, especially when using WR. The authors would like to acknowledge that due to this study being conducted on endurance-trained athletes, these findings may not be applicable to other populations. Furthermore, since most traditional endurance training occurs in a variety of environments, the laboratory conditions used in this study may not be directly comparable to other studies in endurance-trained runners. 

## 5. Conclusions

The present findings indicate that evenly loading the medial and lateral aspect of the calf with WR while running at a speed equivalent to V̇T1 will elicit an increase in metabolic response compared to un-loaded conditions. There is an expected increase in V̇O_2_ and HR response of 2.56% (± 0.75%) and 1.16% (± 0.52%), respectively, for every 1% BM of additional load and an increase in exercise stress of 0.39% (± 0.06%) for the equivalent of 10-min of running for every 1% BM of additional load. A load of at least 1% BM is needed to induce notable increases in V̇O_2_ responses; however, 0.5% BM can produce marked increases in HR responses. These findings provide evidence for guiding minimal loading thresholds and help quantify the potential increase in both V̇O_2_ and HR responses to lower-leg WR loads during short-term, submaximal running. Attaching WR to the legs enables a running-specific form of resistance training to be incorporated into training. Practitioners may be interested in moving the load distally away from the hip, as this placement seems to have a greater impact on metabolic cost, compared to trunk or thigh loading. However, this evidence is based only on 5-min of running and the effects of longer-duration-loaded running under these conditions are still unknown. It also gives means for quantifying an expected TSS for loaded submaximal running for a given duration.

## Figures and Tables

**Table 1:** Acute oxygen consumption responses to lower-leg-loaded wearable resistance.

Training Load (% BM)	V̇O_2_ (L) Mean (SD)	Effect Size (90% CI)	Rating
0%	3.22 (0.48)	-	-
0.5%	3.28 (0.53)	0.09 (−0.02 to 0.19)	(4/96/0) very likely trivial increase
1%	3.36 (0.59)	0.22 (0.9 to 0.34)	(60/40/0) possible small increase
1.5%	3.39 (0.56)	0.28 (0.17 to 0.39)	88/12/0) likely moderate increase
2%	3.39 (0.53)	0.3 (0.19 to 0.40)	(94/6/0) likely moderate increase
2.5%	3.43 (0.59)	0.34 (0.22 to 0.44)	(97/3/0) very likely large increase
3%	3.52 (0.54)	0.51 (0.42 to 0.60)	(100/0/0) most likely very large increase

N.B.; CI, Confidence interval. Data represent V̇O_2_ values collected over the final 2-min period of 5-min of submaximal treadmill running at first ventilatory threshold.

**Table 2:** Acute HR responses to lower-leg loaded wearable resistance.

Training Load (% BM)	HR (bpm) Mean (SD)	Effect Size (90% CI)	Rating
0%	150.2 (10.2)	-	-
0.5%	151.6 (9.09)	0.13 (−0.06 to 0.33)	(28/71/1) possible small increase
1%	153.5 (12.1)	0.30 (0.8 to 0.52)	(78/22/0) likely moderate increase
1.5%	154.1 (10.6)	0.35 (0.19 to 0.52)	(94/6/0) likely moderate increase
2%	155.5 (9.84)	0.49 (0.32 to 0.67)	(100/0/0) most likely very large increase
2.5%	156.7 (9.17)	0.60 (0.44 to 0.76)	(100/0/0) most likely very large increase
3%	156.8 (7.95)	0.62 (0.4 to 0.84)	(100/0/0) most likely very large increase

Abbreviations: CI, Confidence interval. Values are mean HR collected over the final 2-min period of 5-min of submaximal treadmill running at first ventilatory threshold.

**Table 3:** Acute La responses to lower-leg loaded wearable resistance.

Training Load (% BM)	La (mmol/L) Mean (SD)	Effect Size (90%CI)	Rating
0%	1.89 (0.60)	-	-
0.5%	2.29 (0.89)	0.49 (0.3 to 0.95)	(86/13/1) likely moderate increase
1%	2.35 (0.72)	0.63 (0.22 to 1.03)	(96/4/0) very likely large increase
1.5%	2.37 (1.11)	0.45 (−0.03 to 0.93)	(82/16/2) likely moderate increase
2%	2.44 (0.95)	0.65 (0.12 to 1.19)	(92/7/1) likely moderate increase
2.5%	2.61 (0.66)	0.96 (0.52 to 1.39)	(100/0/0) most likely very large increase
3%	2.83 (1.22)	1.05 (0.6 to 1.51)	(100/0/0) most likely very large increase

N.B. CI, Confidence interval; Data represent blood La accumulation values sampled immediately post 5-min of submaximal treadmill running at first ventilatory threshold.

**Table 4:** Acute RPE responses to lower-leg loaded wearable resistance.

Training Load (% BM)	Rate of Perceived Exertion (RPE) Mean (SD)	Effect Size (90% CI)	Rating
0%	2.53 (0.88)	-	-
0.5%	3.03 (1.22)	0.40 (0.07 to 0.72)	(85/15/0) likely moderate increase
1%	3.27 (1.07)	0.63 (0.35 to 0.90)	(99/1/0) very likely large increase
1.5%	3.40 (1.02)	0.73 (0.35 to 1.11)	(99/1/0) very likely large increase
2%	4.00 (1.28)	1.11 (0.78 to 1.43)	(100/0/0) most likely very large increase
2.5%	4.30 (1.15)	1.30 (1.05 to 1.55)	(100/0/0) most likely very large increase
3%	4.53 (1.59)	1.38 (0.98 to 1.79)	(100/0/0) most likely very large increase

N.B. CI, Confidence interval. Data represent RPE scores recorded immediately post 5-min of submaximal treadmill running at first ventilatory threshold.
